# CD8^+^ Regulatory T Cells, and Not CD4^+^ T Cells, Dominate Suppressive Phenotype and Function after *In Vitro* Live *Mycobacterium bovis-*BCG Activation of Human Cells

**DOI:** 10.1371/journal.pone.0094192

**Published:** 2014-04-08

**Authors:** Mardi C. Boer, Krista E. van Meijgaarden, Simone A. Joosten, Tom H. M. Ottenhoff

**Affiliations:** Department of Infectious Diseases, Leiden University Medical Center, Leiden, The Netherlands; University of Palermo, Italy

## Abstract

*Mycobacterium bovis* bacillus Calmette-Guérin (*M. bovis* BCG), the only currently available vaccine against tuberculosis, has been reported to induce regulatory T cells in humans. The activity of regulatory T cells may not only dampen immunogenicity and protective efficacy of tuberculosis-vaccines, but also hamper diagnosis of infection of tuberculosis, when using immune (e.g. IFNγ-release) assays. Still, in settings of infectious diseases and vaccination, most studies have focused on CD4^+^ regulatory T cells, and not CD8^+^ regulatory T-cells. Here, we present a comparative analysis of the suppressive phenotype and function of CD4^+^ versus CD8^+^ T cells after *in vitro* live BCG activation of human cells. Moreover, as BCG is administered as a (partly) live vaccine, we also compared the ability of live versus heatkilled BCG in activating CD4^+^ and CD8^+^ regulatory T cell responses. BCG-activated CD8^+^ T cells consistently expressed higher levels of regulatory T cell markers, and after live BCG activation, density and (co-)expression of markers were significantly higher, compared to CD4^+^ T cells. Furthermore, selection on CD25-expression after live BCG activation enriched for CD8^+^ T cells, and selection on co-expression of markers further increased CD8^+^ enrichment. Ultimately, only T cells activated by live BCG were functionally suppressive and this suppressive activity resided predominantly in the CD8^+^ T cell compartment. These data highlight the important contribution of live BCG-activated CD8^+^ Treg cells to immune regulation and emphasize their possible negative impact on immunity and protection against tuberculosis, following BCG vaccination.

## Introduction

Tuberculosis (TB), one of the major global health challenges, accounted for 1.3 million deaths in 2012. It is estimated that one-third of the world population is (latently) infected with *Mycobacterium tuberculosis* (*Mtb*) [1]. Containment of the disease is dependent on innate and adaptive immune responses, and though CD4^+^ Th1 (IFNγ)-responses are considered quintessential, definition of immunological correlates of protection remains unresolved.

Active TB disease has been associated with decreases in *Mtb*-specific IL17A-producing CD4^+^ T cells [2] and in multifunctional (IFNγ^+^IL2^+^TNFα^+^) CD4^+^ T cells [3]. Conversely, CD4^+^ T cells single-positive for TNFα were identified as a strong classifier of active disease versus latent infection [4]. For *Mtb*-specific CD8^+^ T cells, a reduction in dual IFNγ^+^IL2^+^-secreting cells in active vs. latent TB [LTBI] [5], as well as changes in memory phenotype [5], [6], have been reported. CD8^+^ T cells preferentially recognized heavily infected cells *in vitro* [7]; and higher *Mtb*-specific CD8^+^ responses correlated with clinical parameters of bacterial load (defined as smear-positive vs. smear-negative TB) [6].

*Mtb*-specific immune responses and mycobacterial growth inhibition can be suppressed by circulating, alveolar and pleural CD4^+^ regulatory T cells (Treg cells) in humans [8]–[10]. Recently, also suppression of T cell cytokine production and proliferation by myeloid-derived suppressor cells in TB patients were described [11]. Although CD8^+^ regulatory T cells have been reported in TB [9] and leprosy [12], [13], they remain generally understudied compared to CD4^+^ Treg cells [14]. CD4^+^ T cells producing IL-10 were shown to hamper clinical diagnosis based on dermal reactivity to mycobacterial purified protein derivative (PPD) in anergic TB patients [15]. In PBMCs isolated from patients with active TB, depletion of CD4^+^CD25^+^ or CD4^+^CD25^+^CD39^+^ T cells increased *Mtb*-specific IFNγ production [8], [16]. Healthy, previously BCG-vaccinated volunteers, who were vaccinated with MVA-85A (modified vaccinia virus Ankara expressing antigen 85A), and who exhibited relatively low responses in antigen 85A-specific IFNγ ELISPOTs, had increased frequencies of circulating CD4^+^CD25^+^Foxp3^+^ cells, compared to high IFNγ-responders [17]. Also, MVA85A-induced production of IL17A was affected by Treg responses [18], [19]. IFNγ- and IL17-responses were enhanced by addition of ARL67156 [19], a chemical inhibitor of CD39 [20], suggesting a population of CD39^+^ cells that actively dampened cytokine production. Thus, (CD4^+^) Treg cells can negatively influence immunity and immune dependent protection, both in natural infection and in vaccination settings.

The only currently available vaccine against TB, *Mycobacterium bovis* bacillus Calmette-Guérin (*M. bovis* BCG), induces CD4^+^ and CD8^+^ T cell responses in new-borns [21]–[23] and protects them from disseminated forms of disease; but it does not induce consistent protection against pulmonary TB, especially in adults [24]. One explanation for this lack of protection is the induction of regulatory T cells by the vaccine [14], [25], amongst other hypotheses [26], [27]. CD4^+^CD25^+^ Treg cells have been found after BCG vaccination of new-borns [28] and adults [29], and CD4^+^CD25^+^-depleted T-cell cultures resulted in lower PPD-stimulated IL-10 levels [28]. We previously demonstrated the presence and strong suppressive activity of CD8^+^ Treg cells among live BCG-stimulated PBMCs of *in vitro* PPD-responsive donors, which were enriched for the markers lymphocyte activation gene-3 (LAG-3) [30] and CD39 [31]. Suppressive activity of CD8^+^ Treg cells could be reversed by blocking CC chemokine ligand 4 (CCL-4) [30], membrane-bound TGFβ (mTGFβ) [32] and CD39 [31]. Still, knowledge about CD8^+^ regulatory T-cells is generally limited compared to CD4^+^ Treg cells.

Furthermore, though multiple mycobacterial-activated Treg subsets, either CD4^+^ or CD8^+^, have been demonstrated in humans, no comparative studies have been performed assessing suppressive capacity of *Mycobacterium-*induced CD4^+^ vs. CD8^+^ T cells. In this study, we compared the suppressive phenotype and function of human BCG-activated CD4^+^ and CD8^+^ T cells. We demonstrate significantly higher expression of regulatory markers on live BCG-activated CD8^+^ T cells, compared to CD4^+^ T cells, and enrichment for CD8^+^ Treg cells within the BCG-activated CD25^+^ T cell compartment. Finally, suppressive Treg activity was dominantly present in live BCG-activated CD8^+^, but not in live BCG-activated CD4^+^ T cells, nor in killed BCG activated T cells.

## Results

### Heatkilled vs. live BCG-activated expression of Treg-cell markers on CD4^+^ and CD8^+^ T cells

**Figure 1:**
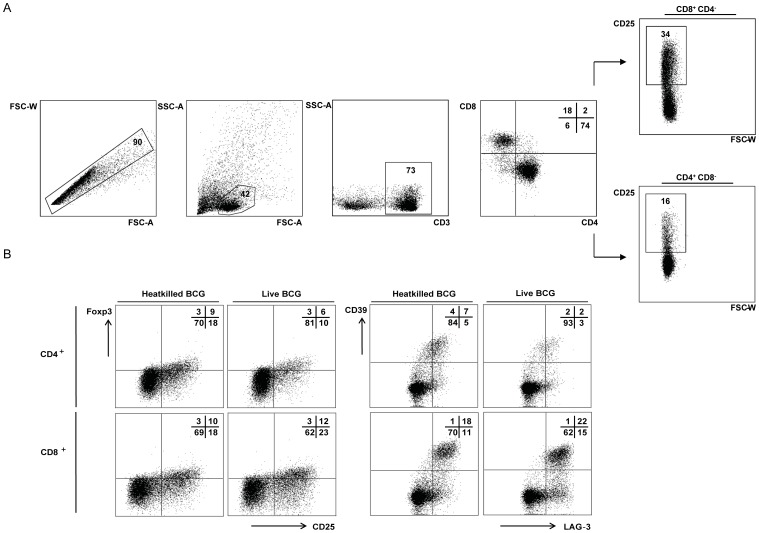
Heatkilled vs. live BCG-activated expression of Treg-cell markers on CD4^+^ and CD8^+^ T cells. A: Gating strategy: cells were gated on single cells, live lymphocytes, CD3^+^ and CD4^+^CD8^−^ vs. CD4^−^CD8^+^. Demonstrated is the synchronized gating on the positive population of interest for CD4^+^CD8^−^ and CD8^+^CD4^−^ T cells; here the CD25-positive population. B: Heatkilled and live BCG activate CD25^+^Foxp3^+^ and LAG-3^+^CD39^+^ T cells. Expression of regulatory T cell markers on CD4^+^ and CD8^+^ T cells of *in vitro* PPD responders was analysed by flowcytometry six days after heatkilled or live BCG stimulation. For each donor gating was compared to samples not stimulated with BCG (demonstrated in Fig. S1). Data are representative of seven responders.

PBMCs were isolated from healthy human donors that had been selected based on their *in vitro* response to mycobacterial PPD as described before [30], [31], [33]. The PBMCs were stimulated with heatkilled or live BCG, and CD4^+^ and CD8^+^ T cells were analysed for regulatory T cell marker expression after six days. Figure 1A depicts the full gating strategy, and an example of the synchronized gating on a positive population for CD4^+^ and CD8^+^ T cells, in compliance with MIATA guidelines [34]. Background expression of Treg-cell markers was compared between CD4^+^ and CD8^+^ populations of samples that were not stimulated with BCG (Figure S1); only the background expression of CCL4 on CD8^+^ T cells was significantly higher compared to CD4^+^ T cells (median 11% vs. 2%; *p* < 0.01; Wilcoxon signed ranks-test) [34]. Heatkilled, as well as live BCG stimulation, activated expression of regulatory T cell markers on CD4^+^ and CD8^+^ T cells of *in vitro* PPD-responsive donors, including CD25, Foxp3, LAG-3 and CD39 (Fig. 1B).

### Treg-cell marker frequency and density are increased on live BCG-activated CD8^+^ vs. CD4^+^ T cells

**Figure 2:**
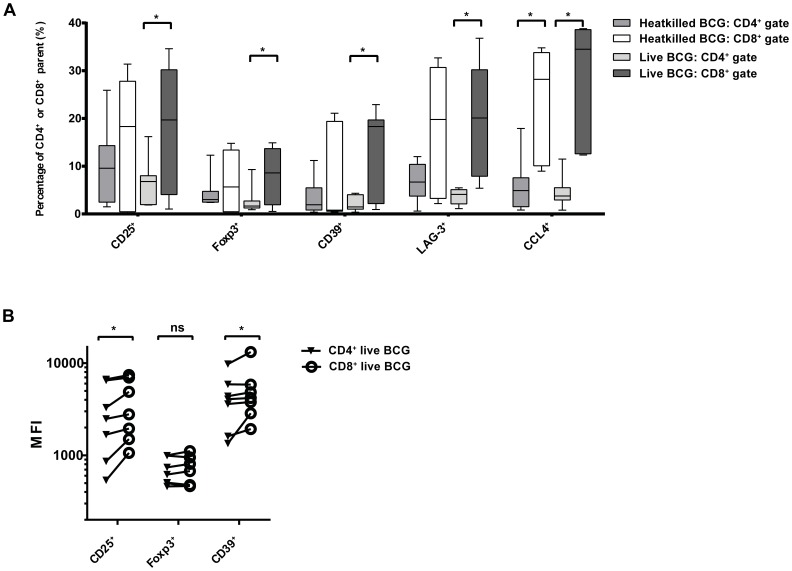
Treg-cell marker frequency and density are increased on live BCG-activated CD8^+^ vs. CD4^+^ T cells. A: BCG induces Treg-cell marker expression on CD4^+^ and CD8^+^ T cells; after live BCG stimulation the percentage of total CD8^+^ T cells expressing CD25, Foxp3, CD39, LAG-3 or CCL4 is significantly higher compared to CD4^+^ T cells, depicted here as frequency of CD8^+^ or CD4^+^ population. Differences in Treg marker expression between heatkilled BCG–activated CD8^+^ vs. CD4^+^ T cells were not significant, except for expression of CCL4; CCL4 expression was also significantly higher on CD8^+^ T cells compared to CD4^+^ T cells in samples not stimulated with BCG (Fig. S1) (*p < 0.05, Wilcoxon signed-ranks test). B: Mean fluorescence intensities (MFIs) of CD25 and CD39 are increased on live BCG-activated CD8^+^ T cells as compared to CD4^+^ T cells. Gating was performed as demonstrated in figure 1A. To assess differences in intrinsic intensity of expression on CD4^+^ and CD8^+^ T cells, respectively, MFIs of positive Treg marker populations in samples not stimulated with BCG were compared; this was similar on CD4^+^ and CD8^+^ T cells for MFIs of CD25, Foxp3 and CD39. Data are representative of seven *in vitro* PPD-responders six days after heatkilled or live BCG stimulation (*p < 0.05; Wilcoxon signed-ranks test).

Heatkilled and live BCG activated a higher percentage of total CD8^+^ T cells, compared to CD4^+^ T cells, that expressed CD25, Foxp3, CD39, LAG-3 or CCL4, depicted in figure 2A as frequency of (CD8^+^ or CD4^+^) parent. Live BCG-activated CD8^+^ T cells exhibited significantly increased Treg-cell marker frequencies compared to live BCG-activated CD4^+^ T cells (*p < 0.05; Wilcoxon signed-ranks test).

To determine cellular densities of expression of Treg-cell markers, mean fluorescence intensities (MFIs) of positive populations were compared for BCG-activated expression of CD25, Foxp3 and CD39. MFIs of CD25 and CD39 were significantly higher on live BCG-stimulated CD8^+^ T cells, compared to CD4^+^ T cells (Fig. 2B; *p*  =  0.02 and *p*  =  0.03, respectively; Wilcoxon signed-ranks test), whereas MFIs of heatkilled BCG-activated CD4^+^ T cells did not differ from heatkilled BCG-activated CD8^+^ T cells (data not shown).

### Co-expression of multiple Treg-cell markers enriches for CD8^+^, and not CD4^+^ T cells

**Figure 3:**
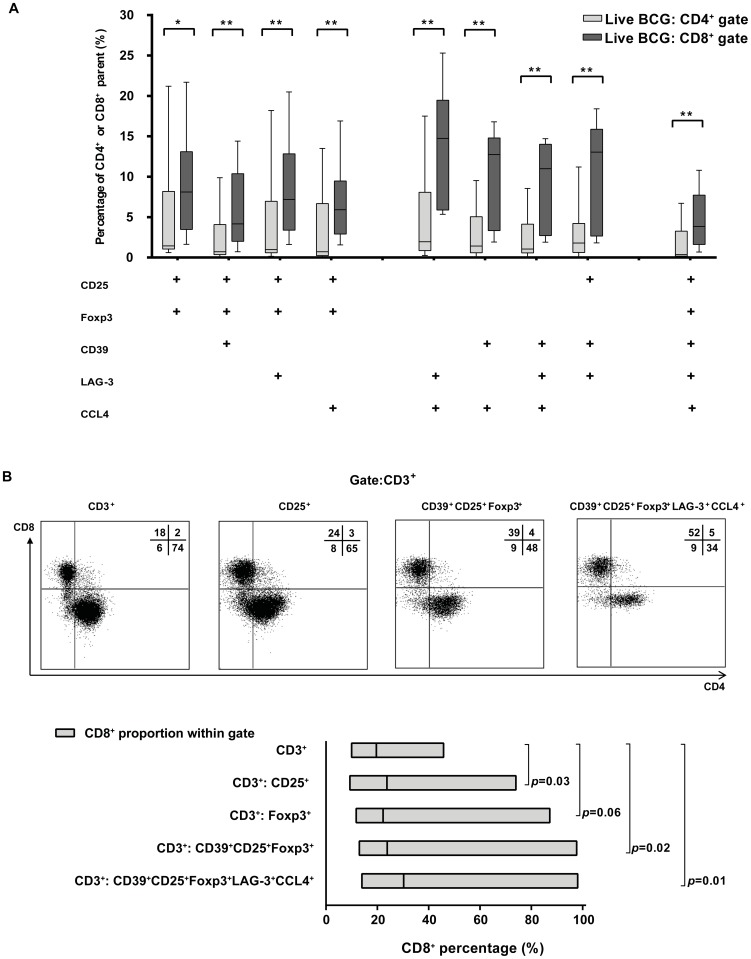
Co-expression of multiple Treg-cell markers enriches for CD8^+^, and not CD4^+^ T cells. A. The percentage of total CD8^+^ T cells co-expressing CD39, LAG-3, CCL4, CD25 and/or Foxp3 in different combinations is significantly increased, compared to CD4^+^ T cells. Demonstrated is a combined analysis using Boolean gating of cells from ten donors six days after live BCG infection. Gating was performed as in figure 1A. Boxes: 25th to 75th percentiles; line at median; whiskers: minimum to maximum (*p < 0.05, **p < 0.01; Wilcoxon signed-ranks test). B. Combining Treg markers enriches for CD8^+^ T cells as opposed to CD4^+^ T cells. Boolean gating was performed on CD3^+^ T cells of ten donors; CD8 vs. CD4 gating is demonstrated (top) and the CD8^+^ proportion of these gated populations is demonstrated (bottom) for a selection of CD3^+^ Boolean gates. The CD8^+^ proportion increased significantly using a combination of Treg markers as compared to the complete CD3^+^ population. Boxes: minimum to maximum, line at median (Wilcoxon signed-ranks test).

Co-expression of multiple Treg-cell markers by live BCG-induced T cells was analysed using Boolean gating (Fig. 3A). A significantly higher percentage of total CD8^+^ T cells was CD25^+^Foxp3^+^, compared to CD4^+^ T cells (*p*  =  0.02; Wilcoxon signed-ranks test). Also, the percentage of total CD8^+^ T cells co-expressing CD25, Foxp3, CD39, LAG-3 and/or CCL4 in various combinations was significantly higher compared to CD4^+^ T cells (*p* < 0.01, Wilcoxon signed-ranks test).

To determine the relative distribution of CD4^+^ and CD8^+^ T cells within the Treg-cell marker positive T cells, we applied Boolean gating to the total CD3^+^ population (Fig. 3B, upper panel), and the CD8^+^ proportion was calculated for the CD3^+^ Boolean gates (Fig. 3B, lower panel). Gating BCG-activated T cells on expression of CD25 or Foxp3, enriched for CD8^+^ T cells compared to the total CD3^+^ population (*p*  =  0.03 and *p*  =  0.06, respectively; Wilcoxon signed-ranks test). Increasing selection of total BCG-activated T cells on regulatory T cell markers further enriched for CD8^+^ T cells significantly (*p*  =  0.01 for CD25^+^Foxp3^+^CD39^+^LAG-3^+^CCL4^+^ T cells, Wilcoxon signed-ranks test).

### Suppressive activity resides predominantly in live BCG-activated CD8^+^ T cells

**Figure 4:**
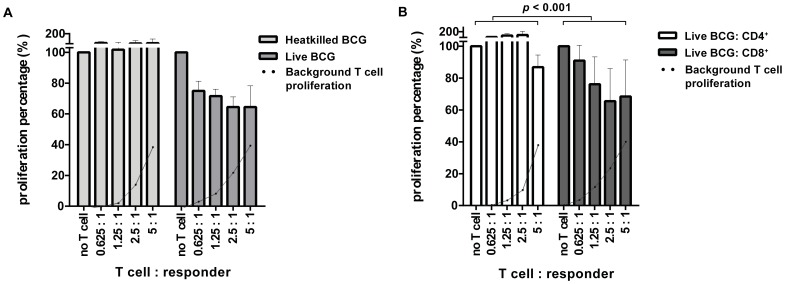
Suppressive activity resides predominantly in live BCG-activated CD8^+^ T cells. Heatkilled BCG-activated and live BCG-activated T cell lines were expanded, and live BCG T-cell lines were enriched for CD4 or CD8 expression using magnetic beads. Suppressive capacity was tested in a co-culture assay by titrating these T cell lines onto a Th1 reporter clone that was stimulated with its cognate peptide [30], [31]. Proliferation was measured by (3H)TdR incorporation after three days. Proliferation was divided by Th1 reporter clone proliferation in the absence of Treg cells to obtain relative proliferation as described previously [30]–[32]. Dotted lines represent background proliferation of T cells, in the absence of reporter clone peptide, relative to Th1 clone proliferation. A. Suppressive activity was confined to live BCG-activated T cells, and could not be demonstrated for heat-killed BCG-specific T cells. Data are depicted as mean + SE of five different heat-killed BCG-activated T cell lines, and six live BCG-activated T cell lines. B. Suppressive activity resides predominantly in CD8^+^ T cells, and not in CD4^+^ T cells (mean + SE of CD4^+^ and CD8^+^ T cell lines of three donors, tested in at least two independent assays; Wilcoxon signed-ranks test, *p* < 0.001).

T cell lines were tested for their capacity to suppress proliferation of an unrelated CD4^+^ T helper-1 clone. This responder clone recognizes a cognate peptide presented in the context of HLA-DR3 in an assay which has been previously reported and validated [30]–[32], [35]. Heatkilled BCG-activated T cells did not suppress proliferation of the responder clone. In contrast, live BCG-stimulated T cells exhibited suppressive activity towards the same responder clone (Fig. 4A). CD8^+^/CD4^+^ ratios of heatkilled BCG-stimulated and live BCG-stimulated T cell lines were 0.06 and 0.1, respectively (Figure S2), suggesting a potential association of T cell subset distribution with suppressive function.

We next separated live BCG-activated T-cell lines into CD4^+^ or CD8^+^ expressing populations using magnetic beads (purity ≥ 97% as assessed by flowcytometry), and tested live BCG-activated CD4^+^ T cells in parallel with live BCG-activated CD8^+^ T cells for their suppressive capacity. Live BCG-activated CD8^+^ T cells suppressed T helper-1 clone proliferation, whereas live BCG-activated CD4^+^ T cells did not significantly inhibit proliferation. Thus, suppressive activity was dominant in live BCG-activated CD8^+^ T cells, compared to live BCG-activated CD4^+^ T cells (Fig. 4B; *p* < 0.001; Wilcoxon signed-ranks test).

## Discussion

In this study, we present a comparative analysis of the suppressive phenotype and function of BCG-activated CD4^+^ vs. CD8^+^ T cells. CD8^+^ T cells consistently expressed higher levels of regulatory T cell markers compared to CD4^+^ T cells; also the cellular density of expression and co-expression of these markers were significantly higher. Selection of T cells based on CD25-positivity after live BCG-activation also enriched for CD8^+^ T cells, and further selection on co-expression of combined regulatory markers further supported CD8^+^ enrichment. Suppressive Treg activity was dominantly present in live BCG- but not heatkilled BCG-activated T cells; finally, the suppressive activity largely resided in the CD8^+^ T cell- and not the CD4^+^ T cell-population.

Multiple CD4^+^ Treg-cell marker expressing subsets have been demonstrated in patients with tuberculosis [9], [10] and after vaccination with MVA85A [18] and BCG [29]. We previously demonstrated the suppressive activity of CD8^+^LAG-3^+^CCL4^+^ and CD8^+^CD39^+^ Treg cells, isolated from live BCG-stimulated PBMCs; in those studies, we also observed upregulation of these markers in the CD4^+^ compartment [30], [31]. However, also non-suppressive, activated human CD4^+^ T cells may transiently upregulate Foxp3-expression such that *in vitro*-induced Foxp3 expression by human CD4^+^ T cells is not necessarily associated with suppressive function [36]. The co-expression of multiple Treg-cell markers can more reliably and specifically identify human Treg cells. In the current study we found that more stringent selection of total BCG-activated T cells using multiple Treg cell markers further enriched for CD8^+^ T cells significantly. In other work using allo-antigen induction of Treg cells by plasmacytoid dendritic cells, also discrepant activation of CD8^+^ vs. CD4^+^ Treg cells has been reported: suppressive activity was mediated by CD8^+^LAG-3^+^Foxp3^+^CTLA-4^+^ T cells, but not by plasmacytoid dendritic cell-induced CD4^+^ T cells [37]. However, no systematic comparative studies have been performed so far comparing suppressive capacity of *Mycobacterium*-induced CD4^+^ vs. CD8^+^ T cells.

The type of antigen used for *in vitro* restimulation of specific responses may significantly influence the results, as stimulation with live mycobacteria could activate significantly different populations of T cells as compared to killed mycobacteria or protein isolates like PPD. CD4^+^ Treg cells have been isolated from PBMCs of active TB patients through ex vivo selection on co-expression of CD4 and CD25 [9], [16], [38], and have been phenotyped after culturing PBMCs with TB-specific peptides [16] or mycobacterial PPD [9]. PPD-stimulated PBMCs of TB patients revealed expansion of CD4^+^CD25^+^Foxp3^+^ T cells in active TB patients, but low numbers of CD8^+^CD25^+^Foxp3^+^ T cells [9]. In the current study, we compared live and heatkilled BCG, where heatkilled BCG was considered to be a primary stimulus for CD4^+^ T cells, through the MHCII-antigen presentation pathway, resembling PPD. It is intriguing, as demonstrated here, that CD8^+^ Treg activity is specifically induced by live as opposed to heatkilled BCG, suggesting that the MHCI-antigen presentation pathway is involved in the activation of these cells, and also that cross-presentation of killed bacteria to CD8^+^ T cells is likely to be insufficient. We hypothesize that BCG, as a live intracellular bacterium, is able to modify antigen presentation/stimulation, although the mechanisms and pathways involved remain unknown at this stage.

The relatively long persistence of BCG as a live intracellular bacterium in the human body after vaccination, as opposed to other vaccines, may be responsible for inducing increased CD8^+^ (regulatory) T cell responses over time, compared to CD4^+^ T cells. Dendritic cells in the skin could optimally cross-present [39] extracellular fragments of BCG after vaccination, further adding to CD8^+^ T cell priming by late cross-presentation. Additional research is needed to clarify the BCG-specific induction of Treg cells *in vivo* and to compare the magnitude and persistence of CD4^+^ and CD8^+^ T cells prospectively, both early after BCG vaccination as well as at later time points.

Studies analysing immune responses induced by *Mtb*-infection, TB disease or BCG vaccination, may have largely overlooked the presence and role of CD8^+^ Treg cells, which may be surprising, considering the initial identification of suppressor cells as CD8^+^ T cells [40], and the early cloning of CD8^+^ suppressor T cells in mycobacterial disease [12], [13]. Immune based diagnosis of TB infection, such as tuberculin skin tests and IFNγ-release assays (IGRAs), vaccine immunogenicity, and perhaps also vaccine induced protection could all be negatively impacted upon by Treg activity [15]–[17]. More research into the induction and activity of Treg cells, and comparative analyses of subsets, could be important to optimal vaccine design as well as a better understanding of correlates of protection. Our study highlights the important contribution of live BCG-activated CD8^+^ Treg cells to immune regulation, and emphasizes the possible negative impact of human CD8^+^ regulatory T cells on immunity to mycobacterial infection and vaccination.

## Materials and Methods

### Ethics Statement

All donors had signed consent for scientific use of blood products. Blood products were collected anonymously, which, according to institutional ethical policy, does not require a separate review by the Ethical Committee.

### Blood Samples

Anonymous buffy coats were collected from healthy adult blood bank donors (Sanquin, Leiden). PBMCs were isolated by density centrifugation and cryopreserved in fetal calf serum-supplemented medium according to Standard Operating Procedure [41]. Cells were counted using the CASY cell counter (Roche, Woerden, The Netherlands). Donors were selected on recognition of mycobacterial PPD by assessing IFNγ production *in vitro*. PBMCs were stimulated with 5 μg/ml PPD (Statens Serum Institute, Copenhagen, Denmark) for 6 days and supernatants were tested in IFNγ-ELISA (U-CyTech, Utrecht, The Netherlands). Positivity was defined as IFNγ production ≥150 pg/ml.

### Cell Cultures and BCG Infection

PBMCs were thawed (64% median viable cell yield) and stimulated with Bacillus Calmette-Guerin (Pasteur). BCG was grown in 7H9 plus ADC, frozen in 25% glycerol and stored at −80°C. Before use, bacteria were thawed and washed in PBS/0.05% Tween 80 (Sigma-Aldrich, Zwijndrecht, The Netherlands). Infections were done at a multiplicity of infection (MOI) of 1.5. For heat-killed BCG stimulated cell cultures, bacteria were inactivated at 80 °C for 30 minutes. PBMCs were cultured for six days in Iscove's modified Dulbecco's medium (Life Technologies-Invitrogen, Bleiswijk, The Netherlands) supplemented with 10% pooled human serum. Sera were pretested in standardized protocols; only sera were pooled that had no inhibitory activity in standard mixed allogeneic lymphocyte cultures. IL-2 (25 U/ml; Proleukin; Novartis Pharmaceuticals UK Ltd., Horsham, UK) was added after 6 days of culture. CD4^+^ and CD8^+^ T cells were enriched by positive selection using magnetic beads (MACS, Miltenyi Biotec, Teterow, Germany). Purity of sorts was ≥ 97% as assessed by flowcytometry.

Restimulation of CD4^+^ cell lines was done in 24 well plates (2x10^5^ cells/w) with αCD3/CD28 beads (Dynabeads Human T-activator, Life Technologies-Invitrogen) and IL-2 (25 U/ml). Pooled, irradiated (30 Gy) PBMCs were added as feeders (1x10^6^ cells/w). CD8^+^ cell lines were restimulated in 96 well roundbottom plates (1x10^4^ cells/w) with αCD3/CD28 beads, IL-2 (50 U/ml), IL-7, IL-15 (both 5 ng/ml, Peprotech, Rocky Hill, NJ, USA) and pooled, irradiated (30 Gy) PBMCs added as feeders (5x10^4^ cells/w). Cells were maintained in IL-2 (100 U/ml).

### FACS Analysis

PBMCs were incubated overnight with αCD3/28 beads, for the last 16 hours Brefeldin A (3 μg/ml, Sigma-Aldrich) was added. The following antibodies were used for surface staining: CD8-HorizonV500 (clone RPA-T8), CD3-PeCy5 (clone UCHT-1), CD4-PerCPCy5.5 (clone RPA-T4) (all BD Biosciences, Eerembodegem, Belgium), and CD39-PE (clone A1; Biolegend, London, U.K.). For intracellular staining we used the FIX&PERM Cell Permeabilization Kit from An Der Grub BioResearch GMBH (Susteren, The Netherlands) and the following antibodies: CCL4-FITC (clone 24006; R&D Systems, Abingdon, UK), Foxp3-Alexa Fluor 700 (clone PCH101; eBioscience, Hatfield, UK), CD25-allophycocyanin-H7 (clone M-A251; BD Biosciences) and LAG-3-atto 647N (clone 17B4; ENZO Life Sciences, Antwerp, Belgium). Samples were acquired on a BD LSRFortessa using FACSDiva software (version 6.2, BD Biosciences) using compensated parameters.

Analysis was performed using FlowJo software (version 9.5.3, Treestar, Ashland, OR, USA). The detailed gating strategy is demonstrated in figure 1A. Cut-off for positive populations of interest was defined by comparison to samples of cell lines not stimulated with BCG (Figure S1) and were similar for CD4^+^ and CD8^+^ T cell populations, as shown in figure 1A. Also, to assess differences in intrinsic frequency and density (MFI) of Treg-cell marker expression on CD4^+^ vs. CD8^+^ T cells, positive Treg marker populations on CD4^+^ and CD8^+^ T cells were compared in samples not stimulated with BCG.

### Suppression Assays

Cell lines were tested for their capacity to inhibit proliferation of a Th1 responder clone (Rp15 1-1) in response to its cognate *M. tuberculosis* hsp65 p3–13 peptide presented by HLA-DR3 positive, irradiated (20 Gy) PBMCs. Proliferation was measured after three days of co-culture by addition of (3H)thymidine (0.5 μCi/well) and incorporation was assessed after 18 hours. Background proliferation was assessed by adding T cells without the peptide. Validation of this co-culture assay has been reported previously [30]–[32], [35].

Proliferation was divided by Th1 reporter clone proliferation in the absence of Treg cells to obtain relative proliferation and enable analysis across experiments. Values represent means from triplicate wells, that were subsequently averaged for repeated experiments per donor. Demonstrated values represent pooled data from different donors. Raw data can be provided per request.

### Statistical Analyses

Wilcoxon signed ranks tests were performed using GraphPad Prism (version 6, GraphPad Software, San Diego, CA, USA) and SPSS statistical software (version 20, SPSS IBM, Armonk, NY, USA).

### Laboratory

Studies were conducted in a laboratory guided by exploratory research principles with established Standard Operating Procedures [41].

## Supporting Information

Figure S1 **Treg-cell marker expression in samples not stimulated with BCG.** Positive populations for Treg-cell markers were defined by comparison with not BCG-stimulated samples for each donor. In the latter samples, only CCL4 expression was significantly higher on CD8^+^ T cells, compared to CD4^+^ T cells.
(TIF)

Figure S2 **CD8^+^: CD4^+^ ratio after expansion of T-cell lines.** Heatkilled and live BCG-activated T cell-lines were expanded and CD4^+^ and CD8^+^ frequencies were assessed by flowcytometry. The median pre-expansion CD8^+^: CD4^+^ ratio was 0.3.
(TIF)
