# Supplementary figures and images for: CD8+ Regulatory T Cells, and Not CD4+ T Cells, Dominate Suppressive Phenotype and Function after In Vitro Live Mycobacterium bovis-BCG Activation of Human Cells
Source: PLoS One. 2014 Apr 8;9(4):e94192. doi: 10.1371/journal.pone.0094192 (PMC3979753; doi:10.1371/journal.pone.0094192)

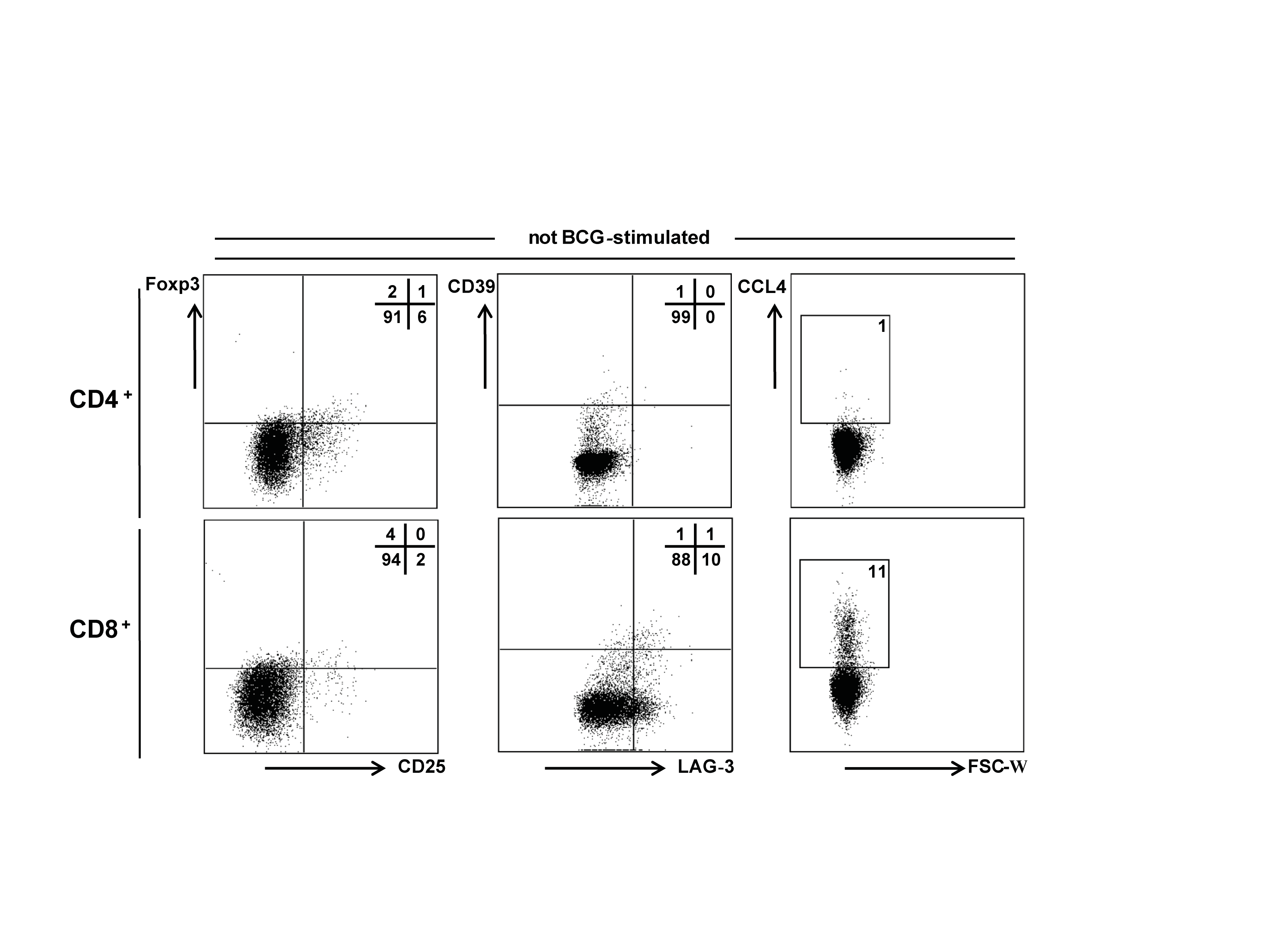

Supplement: Figure S1 — Treg-cell marker expression in samples not stimulated with BCG. Positive populations for Treg-cell markers were defined by comparison with not BCG-stimulated samples for each donor. In the latter samples, only CCL4 expression was significantly higher on CD8+ T cells, compared to CD4+ T cells. (TIF) [file pone.0094192.s001.tif]

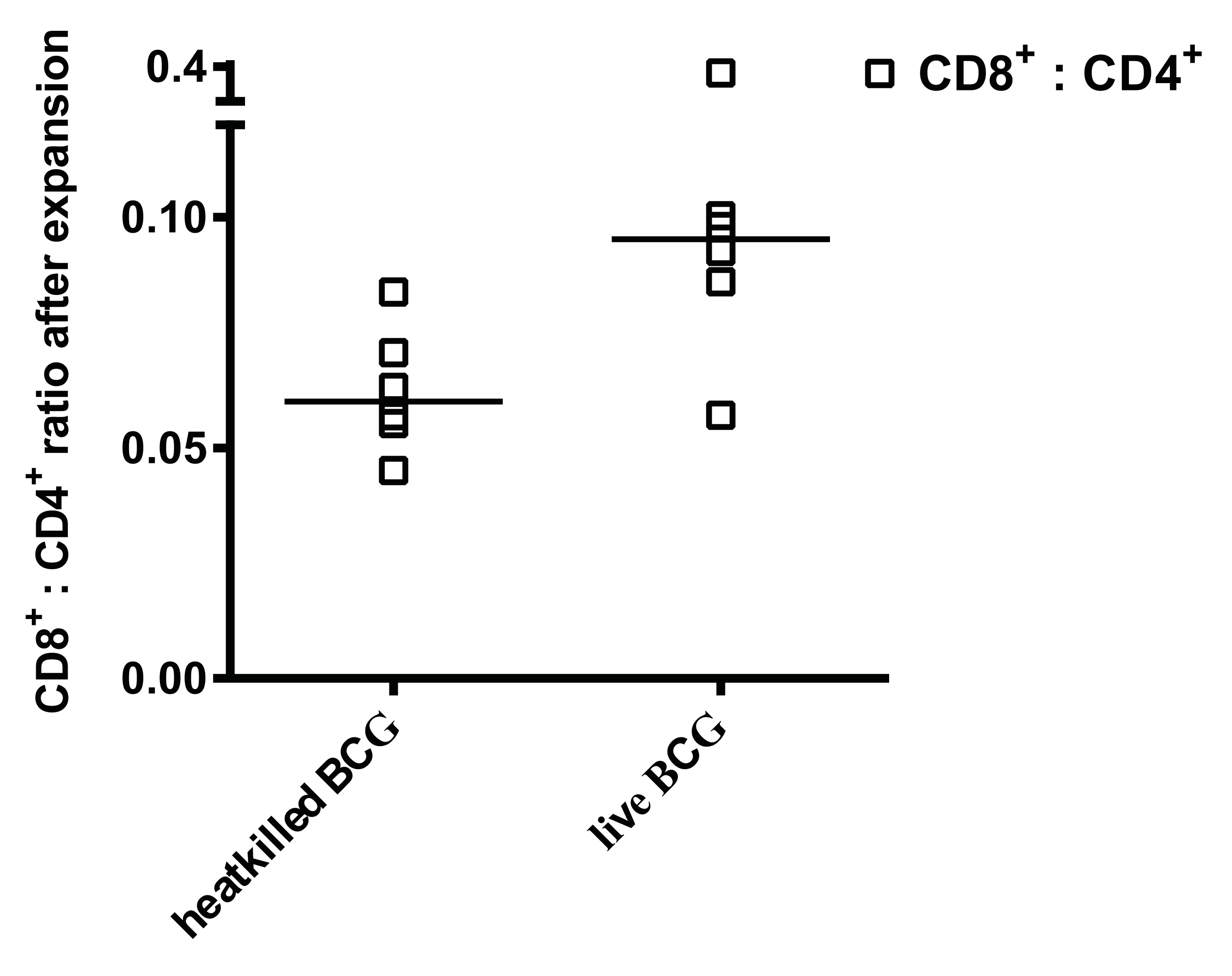

Supplement: Figure S2 — CD8+: CD4+ ratio after expansion of T-cell lines. Heatkilled and live BCG-activated T cell-lines were expanded and CD4+ and CD8+ frequencies were assessed by flowcytometry. The median pre-expansion CD8+: CD4+ ratio was 0.3. (TIF) [file pone.0094192.s002.tif]
